# Sasanquasaponin from *Camellia oleifera* Abel Exerts an Anti-Inflammatory Effect in RAW 264.7 Cells via Inhibition of the NF-κB/MAPK Signaling Pathways

**DOI:** 10.3390/ijms25042149

**Published:** 2024-02-10

**Authors:** Yaxin Zhao, Nanshan Zhao, Larwubah Kollie, Dongfeng Yang, Xiaodan Zhang, Haihua Zhang, Zongsuo Liang

**Affiliations:** Key Laboratory of Plant Secondary Metabolism and Regulation of Zhejiang Province, College of Life Sciences and Medicine, Zhejiang Sci-Tech University, Hangzhou 310018, China; yaxin2017202103@163.com (Y.Z.); 15619000578@163.com (N.Z.); 15619032578@163.com (L.K.); yangdongfeng@zstu.edu.cn (D.Y.); zxd_211@aliyun.com (X.Z.)

**Keywords:** sasanquasaponin, anti-inflammation, molecular mechanisms, camellia seeds

## Abstract

Sasanquasaponin (SQS), a secondary metabolite that is derived from Camellia seeds, reportedly possesses notable biological properties. However, the anti-inflammatory effects of SQS and its underlying mechanisms remain poorly explored. Herein, we aimed to investigate the anti-inflammatory properties of SQS against lipopolysaccharide (LPS)-induced inflammatory responses in RAW264.7 cells, focusing on the nuclear factor-κB (NF-κB) and MAPK signaling pathways. SQS was isolated using a deep eutectic solvent and D101 macroporous adsorption resin and analyzed using high-performance liquid chromatography. The viability of LPS-stimulated RAW264.7 was assessed using the CCK-8 assay. The presence of reactive oxygen species (ROS) was evaluated using 2′,7′-dichlorofluorescein-diacetate. The expression levels of inducible nitric oxide synthase (iNOS), cyclooxygenase-2 (COX-2), tumor necrosis factor-α (TNF-α), interleukin-1β (IL-1β), and interleukin-6 (IL-6) were detected using reverse transcription–quantitative PCR and ELISA. Western blot was performed to analyze the protein expression of LPS-induced RAW264.7 cells. Herein, SQS exhibited anti-inflammatory activity: 30 μg/mL of SQS significantly reduced ROS generation, inhibited the LPS-induced expression of iNOS and COX-2, and attenuated the production of pro-inflammatory cytokines IL-1β, IL-6, and TNF-α. The anti-inflammatory activity was potentially mediated by inhibiting the phosphorylation of IκBα and p65 in the NF-κB signaling pathway and the phosphorylation of ERK and JNK in the MAPK signaling pathway. Accordingly, SQS could inhibit inflammation in LPS-induced RAW264.7 cells by suppressing the NF-κB and MAPK signaling pathways. This study demonstrated the potential application of SQS as an anti-inflammatory agent.

## 1. Introduction

*Camellia oleifera*, originating in China, belongs to the family Theaceae and is a major source of woody edible oil [1]. Camellia oil, known as “oriental olive oil”, is extracted from the seeds of *C. oleifera* and has excellent color, aroma, and taste. This oil has been used in food therapy to prevent cardiovascular diseases such as hypertension, coronary heart disease, and atherosclerosis [2]. In folk medicine, camellia oil is applied topically to treat conditions such as burns, bruises, cuts, scalds, and acute inflammation. *C. oleifera* seed oil and Camellia oil cake (a by-product that is obtained during oil extraction from seeds) are excellent sources of phytochemicals, including Sasanquasaponin (22-O-angeloyl camelliagenin C 3-O-[β-D-glucopyranosyl (1, 2)] [β-D-glucopyranosyl (1, 2)-α-L-arabinopyranosyl (1, 3)]-β-D-glucopyranosiduronic acid, C_58_H_92_O_26_, SQS). SQS is a mixture of oleanocane pentacyclic triterpenoid saponins, with the basic structure comprising three parts: saponins, glycosomes, and organic acids. SQS is known to possess notable biological activity and exert pharmacological effects [3,4]. SQS reportedly exhibits analgesic, antioxidant, antitumor, and burn-reducing effects, and these activities have been attributed to the ability of SQS to suppress inflammatory factors [5,6,7]. Chen et al. [8] have reported that treatment with SQS protected rat cardiomyocytes against oxidative stress that was induced by anoxia–reoxygenation injury. Furthermore, SQS may exert anticancer activity against multiple tumors, including breast and liver cancer [9]. Ye et al. [10] have revealed that SQS from the defatted seeds of *C. oleifera* inhibited the production of pro-inflammatory cytokines and prostaglandin E2 (PGE_2_), thereby attenuating the inflammatory response in carrageenan-induced edema in rats and croton oil-induced ear inflammation in mice. Li et al. [6] have shown that SQS suppresses nitric oxide (NO) and PGE_2_ secretion in lipopolysaccharide (LPS)-stimulated RAW264.7 cells. However, the anti-inflammatory role of SQS and the underlying mechanism mediating this activity are yet to be comprehensively elucidated.

Inflammation is a biological response to various physical and chemical stimuli or microbial toxins that cause tissue damage. It is a normal immune response to external agents. Moderate inflammatory responses are usually beneficial and necessary to protect against harmful stimuli. However, excessive and chronic inflammation (over-stimulation or prolonged inflammatory response) can lead to tissue damage, functional loss, and diseases [11]. The initial inflammation response is significantly associated with the generation of excessive reactive oxygen species (ROS). The disruption of the balance between ROS generation and scavenging results in abnormal ROS levels in the inflammatory microenvironment, thereby resulting in severe cellular damage. This triggers an intracellular inflammatory signaling cascade that ultimately leads to the activation of transcription factors that are responsible for regulating the production of pro-inflammatory cytokines and other effector molecules, such as interleukin (IL)-1β, IL-6, tumor necrosis factor (TNF)-α, inducible NO synthase (iNOS), and cyclooxygenase-2 (COX-2) [12,13,14]. The nuclear factor-κB (NF-κB) and MAPK signaling pathways play a crucial role in regulating the expression of genes that are associated with immune and inflammatory responses [15,16]. The mechanism through which C. oleifera-derived SQS regulates signaling pathways that are involved in mediating anti-inflammatory effects remains unclear. The regulation of macrophage function in inflammation is critical in disease prognosis. Notably, few studies have examined the effect of SQS on macrophages in regulating inflammation. Inflammation-related diseases are common and critical, and developing natural compounds that are capable of suppressing the inflammatory response with fewer side effects is essential to establishing novel anti-inflammatory therapeutic interventions [17]. To confirm the pharmacological effects of SQS, we isolated and purified SQS from *C. oleifera* seeds and evaluated its anti-inflammatory effects and underlying mechanisms in the LPS-induced RAW264.7 cells. This study provides a fundamental basis for the application of SQS in research and development as an anti-inflammatory agent.

## 2. Results

### 2.1. HPLC Analysis

**Figure 1:**
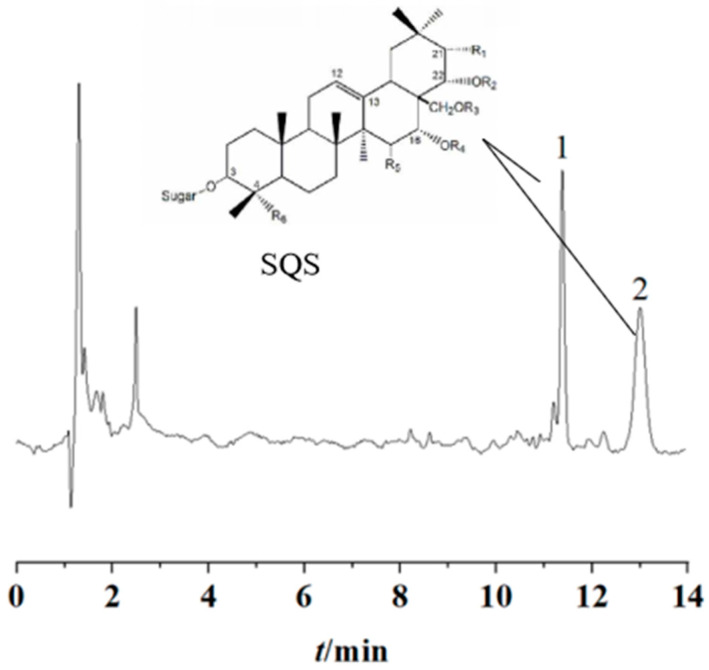
High-performance liquid chromatogram of saponins in *Camellia oleifera* seeds.

SQS that was extracted from *C. oleifera* seed exhibited a single peak in the HPLC chromatograph, and its purity was calculated based on the relative peak area. Peaks 1 and 2 were identified as SQS by co-injection with the standard. The purity of the SQS compounds (tr = 11.381 min and tr = 13.003 min) was 225.33 ng/mL (Figure 1).

### 2.2. Effects of SQS on RAW264.7 Cell Viability

**Figure 2:**
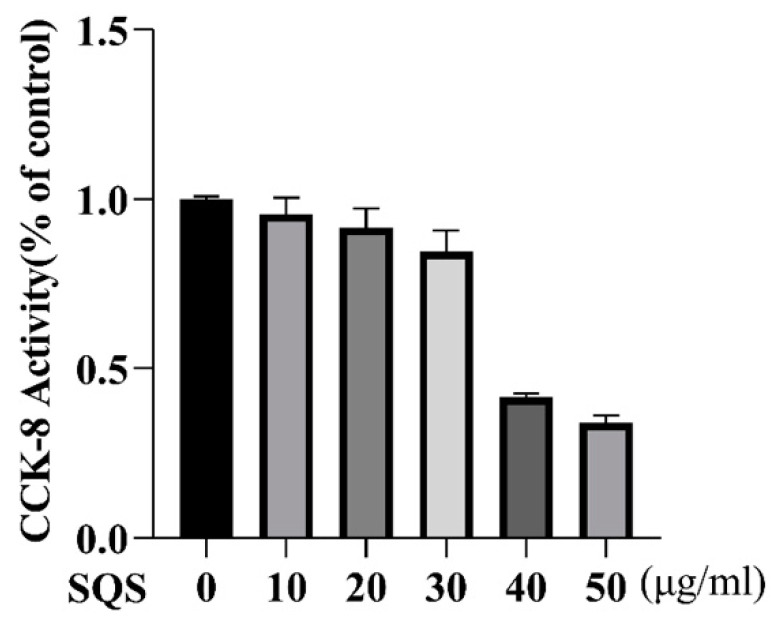
Effect of SQS on the survival of RAW264.7 cells. RAW-264.7 cells were treated with SQS at concentrations of 0–50 μg/mL for 24 h, and cell viability was determined using CCK-8 assay. Data are presented as the mean ± standard deviation (SD) of three independent experiments. LPS, lipopolysaccharide; SQS, sasanquasaponin.

The CCK-8 cell viability assay showed that at concentrations of 10, 20, and 30 μg/mL, SQS did not exert significant cytotoxicity against RAW-264.7; however, 40 and 50 μg/mL of SQS exhibited significant cytotoxic effects against RAW-264.7 cells (Figure 2). Therefore, a concentration range of 10–30 μg/mL was used for subsequent functional studies of SQS.

### 2.3. SQS Reverses LPS-Induced ROS Generation

**Figure 3:**
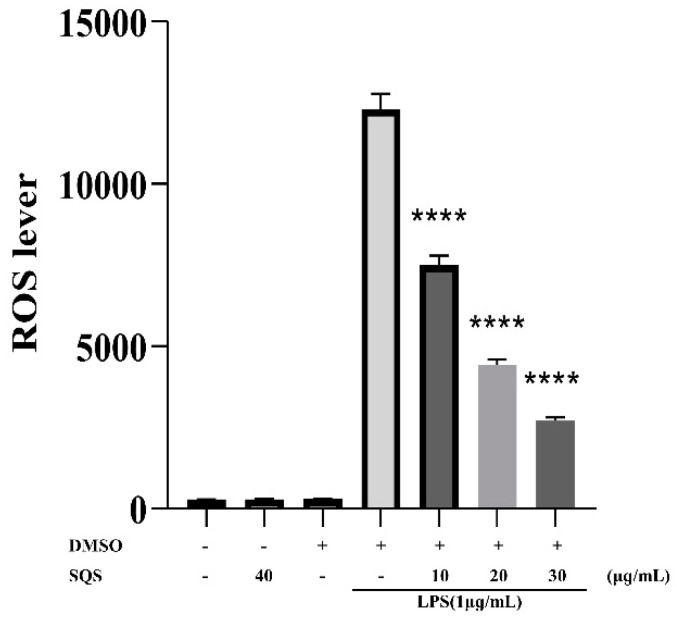
Effects of SQS on LPS-induced ROS production. Data are presented as the mean ± standard deviation (SD) of three independent experiments. **** *p* < 0.0001 compared with LPS-stimulated cells. LPS, lipopolysaccharide; ROS, reactive oxygen species; SQS, sasanquasaponin.

We investigated the effect of SQS on ROS generation in LPS-treated RAW 264.7 macrophages. Treatment with LPS enhanced intracellular ROS levels in macrophages after 3 h. Notably, SQS could significantly reduce the LPS-induced ROS production in a dose-dependent manner. Treatment with 10, 20, and 30 µg/mL SQS significantly (*p* < 0.01) decreased ROS generation by 39, 64, and 78%, respectively, when compared with LPS treatment (Figure 3).

### 2.4. Effects of SQS on LPS-Induced Gene Expression of iNOS and COX-2

**Figure 4:**
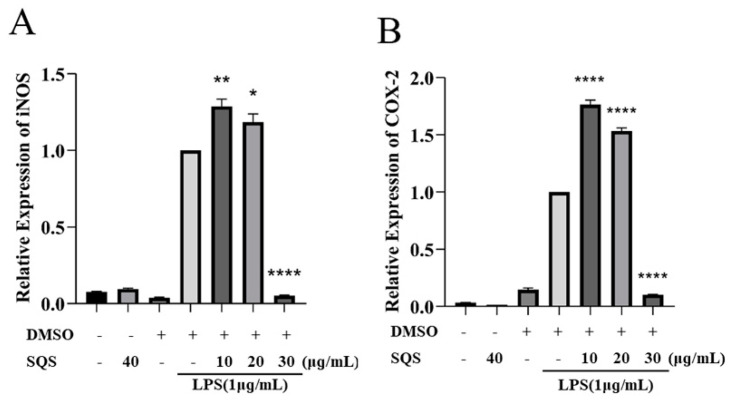
SQS inhibits the LPS-induced transcription of iNOS and COX-2. (**A**,**B**) RAW-264.7 cells were pre-incubated with SQS (0–30 μg/mL) or DMSO for 1 h, followed by stimulation with LPS (1 μg/mL) for 3 h. Transcription of iNOS and COX-2 in the cell lysate was detected using the RT-qPCR method. Each value is the mean ± standard deviation (SD) of at least three independent experiments. * *p* < 0.05, ** *p* < 0.01, **** *p* < 0.0001 compared with LPS-stimulated cells. COX-2, cyclooxygenase-2; DMSO, dimethyl sulfoxide; iNOS, inducible nitric oxide synthase; LPS, lipopolysaccharide; SQS, sasanquasaponin; RT-qPCR, reverse transcription–quantitative PCR.

Stimulating RAW-264.7 cells with LPS is a typical cellular inflammation model, and we successfully constructed this inflammation model to evaluate the anti-inflammatory effects of SQS. The effect of SQS on the mRNA expression of iNOS and COX-2 in LPS-induced RAW264.7 cells was detected using the RT-qPCR method. Herein, we found that the mRNA expression levels of iNOS and COX-2 were significantly increased in the LPS-treated group. Treatment with SQS inhibited the LPS-induced expression of iNOS and COX-2 mRNA in a concentration-dependent manner (Figure 4A,B).

### 2.5. SQS Suppresses the Pro-Inflammatory Gene Expression in LPS-Treated RAW 264.7 Cells

**Figure 5:**
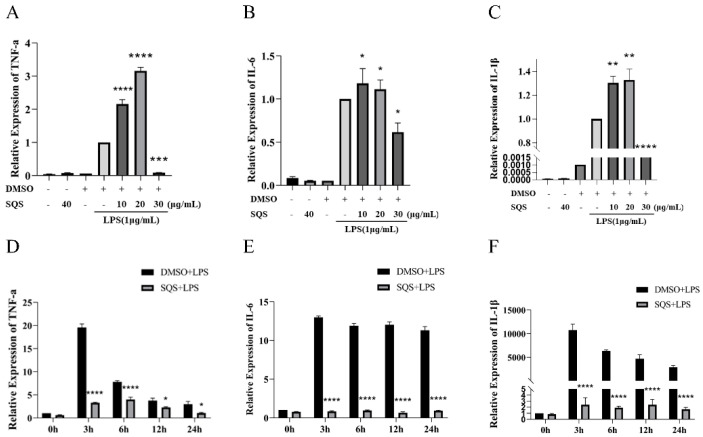
SQS inhibits the transcription of pro-inflammatory cytokines in LPS-stimulated RAW 264.7 cells. (**A**–**F**) RAW-264.7 cells were pre-incubated with SQS (0–30 μg/mL) or DMSO for 1 h, followed by stimulation with LPS (1 μg/mL) for 3 h (**A**–**C**). RAW-264.7 cells were pre-incubated with SQS (30 μg/mL) for 1 h, followed by stimulation with LPS (1 μg/mL) for 3, 6, 12, and 24 h (**D**–**F**). mRNA expression levels of TNF-α, IL-6, and IL-1β in cell lysates were measured using RT-qPCR. Each value represents the mean ± standard deviation (SD) of at least three independent experiments. * *p* < 0.05, ** *p* < 0.01, *** *p* < 0.001, **** *p* < 0.0001 compared with LPS-stimulated cells. IL-1β, interleukin-1β; IL-6, interleukin-6; LPS, lipopolysaccharide; SQS, sasanquasaponin; TNF-α, tumor necrosis factor-α; RT-qPCR, reverse transcription–quantitative PCR.

Based on the RT-qPCR results, 30 μg/mL of SQS significantly inhibited the production of TNF-α (*p* < 0.01) and IL-1β (*p* < 0.01), with inhibition rates of 91.2 and 88.73%, respectively (Figure 5A,B). Simultaneously, 30 μg/mL of SQS also inhibited IL-6 expression, with an inhibition rate of 38.3% (Figure 5C). Notably, SQS regulated cytokine production throughout the LPS incubation period, especially during the early stages (3 h) (Figure 5D–F). Treatment with SQS downregulated the expression levels of TNF-α, IL-6, and IL-1β that were released from RAW-264.7 cells in a time-dependent manner (Figure 5D–F). Based on these findings, SQS exhibited a time-dependent negative regulatory effect on the inflammatory response of LPS-stimulated RAW-264.7 cells at the mRNA level (Figure 5A–F)

### 2.6. SQS Inhibits Pro-Inflammatory Protein Expression in LPS-Treated RAW 264.7 Cells

**Figure 6:**
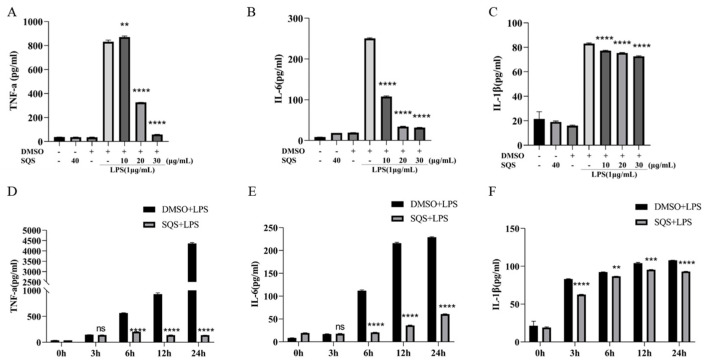
SQS inhibits the expression of pro-inflammatory cytokines in LPS-stimulated RAW 264.7 cells. (**A**–**F**) RAW-264.7 cells were pre-incubated with SQS (0–30 μg/mL) or DMSO for 1 h, followed by stimulation with LPS (1 μg/mL) for 6 h (**A**–**C**). RAW-264.7 cells were pre-incubated with 30 μg/mL of SQS for 1 h, followed by stimulation with LPS (1 μg/mL) for 3, 6, 12, and 24 h (**D**–**F**). Protein expression levels of TNF-α, IL-6, and IL-1β in cell culture supernatants were measured using ELISA. Each value represents the mean ±standard deviation (SD) of at least three independent experiments. ^ns^ *p* > 0.05, ** *p* < 0.01, *** *p* < 0.001, **** *p* < 0.0001 compared with LPS-stimulated cells. DMSO, dimethyl sulfoxide; IL-1β, interleukin-1β; IL-6, interleukin-6; LPS, lipopolysaccharide; SQS, sasanquasaponin; TNF-α, tumor necrosis factor-α.

To determine whether SQS inhibits the expression of pro-inflammatory cytokines in LPS-induced RAW-264.7 cells, we measured the protein levels of three pro-inflammatory cytokines (TNF-α, IL-6, and IL-1β) using ELISA (Figure 6A–F). Herein, SQS significantly reduced the protein levels of TNF-α and IL-6 in a concentration-dependent manner (Figure 6A,B), with no significant inhibitory effect observed on IL-1β expression (Figure 6C). Compared with the LPS treatment, 30 μg/mL of SQS inhibited the expression of TNF-α and IL-6 protein by 92.94 and 87.35%, respectively (Figure 6A,B). Furthermore, SQS regulated cell cytokines throughout the LPS incubation period, especially during the late stage (24 h) (Figure 6D–F). Treatment with SQS downregulated the expression levels of TNF-α, IL-6, and IL-1 β that were released by RAW-264.7 cells in a time-dependent manner (Figure 6D–F). These results indicate that SQS could exert a concentration and time-dependent negative regulatory effect on the pro-inflammatory response of LPS-stimulated RAW-264.7 cells at the protein level (Figure 6A–F).

### 2.7. SQS Exerts Inflammatory Effects by Inhibiting NF-κB and MAPK Signaling

**Figure 7:**
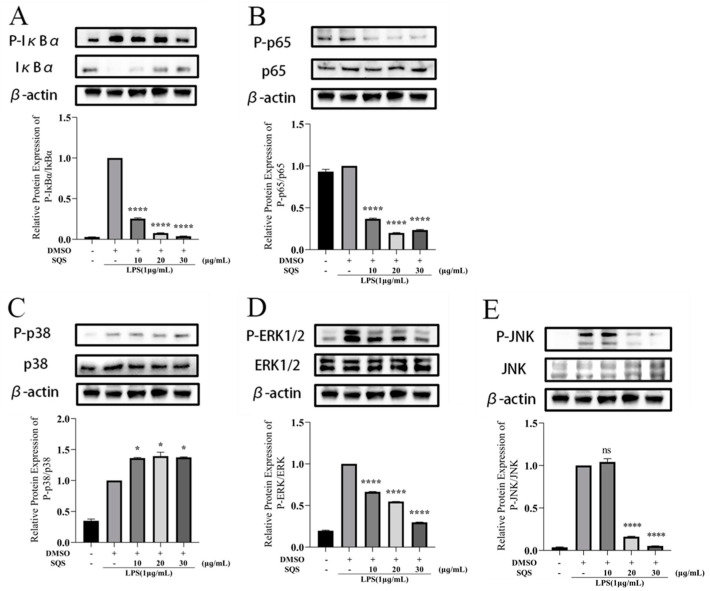
Effect of SQS on LPS-induced NF-κB (**A**,**B**) and MAPK (**C**–**E**) pathways in RAW 264.7 cells. Each value represents the mean ±standard deviation (SD) of at least three independent experiments. * *p* < 0.05, **** *p* < 0.0001 compared with LPS-stimulated cells. LPS, lipopolysaccharide; SQS, sasanquasaponin; NF-κB, nuclear factor-κB.

Given that NF-κB and MAPK signaling are major pathways of inflammatory responses in LPS-stimulated RAW 264.7 macrophages [18], we next analyzed the phosphorylation levels of NF-κB and MAPK signaling proteins after treatment of RAW 264.7 macrophages with LPS or SQS. In the NF-κ B signaling pathway, the phosphorylation of active p65 and IκBα can lead to inflammation. Therefore, it is necessary to further determine the activity of representative proteins p65 and IκBα. As shown in Figure 7A,B, the expression levels of p-p65 and p-IκBα were significantly enhanced in LPS-treated RAW 264.7 cells; the expression levels of total p65 were equal across all groups. However, the expression of IκBα was reduced in the LPS-treated group. Conversely, IκBα expression was enhanced in a concentration-dependent manner in the SQS-treated group. These results indicate that LPS treatment caused IκBα degradation, and SQS inhibited the degradation of IκBα. Treatment with SQS significantly reduced the expression levels of p-IκBα (*p* < 0.0001) and p-p65 (*p* < 0.0001) compared with LPS treatment. Treatment with 10, 20, and 30 μg/mL of SQS inhibited p-IκBα expression by 74.51, 92.31, and 96.17% and p-p65 expression by 63.22, 80.08, and 76.62%, respectively. Collectively, these results indicate that SQS exerted a negative regulatory effect on LPS-induced NF-κB signaling in RAW264.7 cells via the sequential modulation of key events that are involved in the transcription of the pro-inflammatory genes.

In mammalian cells, there are three representative MAPK pathways: ERK, JNK, and the p38 pathway [18,19]; the effect of SQS on the MAPK pathway was evaluated by measuring the expression levels of these kinases. As shown in Figure 7C–E, the levels of ERK, JNK, and p38 did not differ significantly in the LPS-induced cells, while p-ERK, p-JNK, and p-p38 levels increased significantly (*p* < 0.0001). Notably, LPS activated the MAPK signaling pathway via the phosphorylation of ERK, JNK, and p38, triggering an inflammatory response. However, in SQS-treated cells, the levels of p-p38 remained unaltered, and p-ERK and p-JNK levels decreased significantly and in a concentration-dependent manner. Treatment with 10, 20, and 30 μg/mL of SQS inhibited p-ERK expression by 33.60, 45.23, and 70.20% and p-JNK expression by 4.19, 83.87, and 94.69%, respectively. Based on these findings, SQS could significantly block the production of p-ERK and p-JNK in RAW264.7 cells but did not block the production of p-p38 (Figure 7C–E). Therefore, treatment of RAW264.7 cells with SQS downregulated the phosphorylation of ERK and JNK, which are crucial in mediating the MAPK pathway; this finding may be partly responsible for the SQS-mediated anti-inflammatory effect.

## 3. Discussion

Plants contain various secondary metabolites that are extensively used to treat diseases, and these compounds are capable of stimulating biochemical reactions in cells. Therefore, in the current study, we aimed to investigate the anti-inflammatory activity and mechanisms of SQS that is derived from the seeds of C. oleifera. Notably, the detection of ROS levels is crucial in anti-inflammatory experiments, and the measurement of intracellular ROS levels can reflect the extent of oxidative damage in the body. Excessive ROS can provoke oxidative stress, which induces inflammation via the generation of oxidative decomposition products and activation of inflammatory cells to secrete pro-inflammatory factors. Accordingly, there is a close relationship between inflammatory reactions and oxidation. Herein, we found that 10–30 μg/mL of SQS significantly suppressed the secretion of ROS (Figure 3). Based on our findings, the anti-inflammatory effect of SQS may be related to the inhibition of ROS production. To examine the anti-inflammatory activity of natural products, RAW264.7 cells stimulated with LPS to induce an inflammatory response were used as an in vitro cell model [18,19]. LPS binds to toll-like receptor 4 (TLR-4) of RAW264.7 cells and stimulates the production of pro-inflammatory cytokines, such as TNF-α, IL-1 β, and IL-6 by activating intracellular signaling pathways (NF-κB and MAPK signaling pathways) and promoting the formation of representative inflammatory cytokines such as nitric oxide (NO) and PGE_2_ [20]. The inhibition of iNOS activation prevents pathological overproduction of NO, demonstrating therapeutic effects on certain types of inflammation. COX-2, a rate-limiting enzyme for prostaglandin biosynthesis at the site of inflammation, catalyzes the conversion of arachidonic acid to PGE_2_ [21]. TNF-α is involved in the inflammatory response, mediating multiple antitumor and immunomodulation biological processes. IL-6 plays a crucial role in regulating cell growth and differentiation, immune function, hematopoietic function, and anti-inflammatory function. IL-1β is a key pro-inflammatory cytokine that participates in various autoimmune inflammatory responses and cellular activities [22,23,24]. Therefore, determining whether anti-inflammatory substances can reverse the overproduction of iNOS, COX-2, TNF-α, IL-1β, and IL-6 is an effective approach for treating inflammatory diseases. In the current study, treatment with SQS at 10, 20, and 30 μg/mL reduced IL-6, TNF-a, and IL-1β levels in LPS-induced cells in a concentration-dependent manner. Treatment with 30 μg/mL of SQS for 3, 6, 12, and 24 h time-dependently reduced IL-6, TNF-a, and IL-1β levels in the LPS-induced cells. Additionally, the RT-qPCR results revealed that SQS reduced the transcription levels of iNOS and COX-2. These results indicate that SQS exhibits substantial anti-inflammatory activity.

NF-κB is crucial in regulating the production of genes that are associated with immune and inflammatory responses [25,26,27]. Under resting conditions, the main p65(RelA)/p50 dimer is bound to the inhibitory protein (IκBα) of NF-κB, which remains free in the cytoplasmic matrix and cannot be internalized into the nucleus. Upon LPS stimulation, phosphorylation occurs at S32/36 on NF-κBα (IκBα) and S536 on NF-κB p65. Phosphorylation of IκBα at S32/36 induces the degradation of IκBα and disrupts binding to NF-κB, thereby resulting in the translocation of the nuclear localization signal of the NF-kB complex into the nucleus. This initiates the transcription of target genes, including inflammatory cytokines and inflammatory mediators. Therefore, the inhibition of the NF-κB signaling pathway is a promising strategy for treating inflammatory diseases. Notably, several anti-inflammatory saponins have been shown to suppress NF-κB signaling. Tian et al. [28] have revealed that total saponins from the leaves of *Tribulus terrestris* L. exert anti-inflammatory effects by inhibiting the activation of the NF-κB signaling pathway. In addition, Park et al. [29] and Kang et al. [30] have shown that ginseng and Soybean saponins can suppress NF-κB activation, reducing the levels of pro-inflammatory factors. In the current study, LPS induction reduced the intracellular IκBα content. However, the p65 content remained unchanged, whereas levels of p-IκBα and p-p65 were elevated. Notably, SQS-treated cells exhibited elevated levels of IκBα, whereas p-IκBα and p-p65 levels were reduced (Figure 6). These findings are consistent with the reports by Li et al. Therefore, SQS acts on the NF-κB signaling pathway by inhibiting the phosphorylation of IκBα and p65 and protecting IκBα from degradation.

In addition to NF-κB signaling, the MAPK pathway transmits extracellular signals to the cytoplasm and nucleus and is a drug target for inflammatory disease [31]. MAPK is divided into three pathways: ERK 1/2, JNK, and p38 MAPK pathways. When RAW 264.7 macrophages are stimulated by various stress stimuli, including LPS and TLR ligands, MAPK transduces extracellular stimulation signals into the cell and its nucleus, contributing to the increased synthesis of inflammatory mediators in macrophages, consequently enhancing the inflammatory response. Therefore, major events in the MAPK signaling pathway substantially determine the expression of cytokines, chemokines, and other inflammatory mediators. Furthermore, we confirmed that LPS can enhance the activity of p-p38, p-ERK, and p-JNK in RAW264.7 cells. However, pre-treatment with SQS significantly decreased the activation of these kinases (including p-ERK and p-JNK) in LPS-stimulated cells. Additionally, Su et al. [24] have confirmed that saponins from *Aster tataricus* can exert an anti-inflammatory effect by inhibiting the activation of the MAPK signaling pathway, which is consistent with our findings. Therefore, a functional link exists between MAPK regulation and SQS activity.

**Figure 8:**
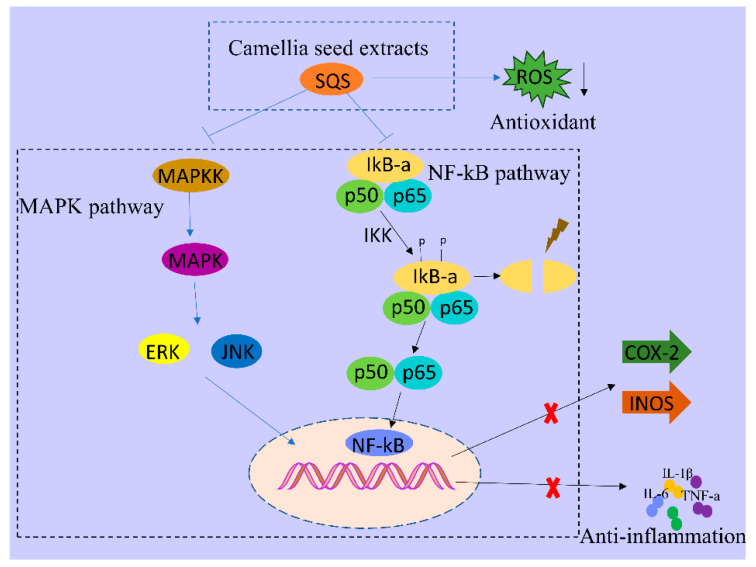
Anti-inflammatory effect of SQS. SQS can protect cells from inflammatory damage by reducing the production of pro-inflammatory factors by suppressing ROS production and inhibiting the activity of COX-2 and iNOS, thereby exhibiting anti-inflammatory behavior by suppressing NF-κB and MAPK signaling pathways. COX-2, cyclooxygenase-2; iNOS, inducible nitric oxide synthase; SQS, sasanquasaponin; ROS, reactive oxygen species.

Natural products can directly or indirectly alter the structure and expression of genes that are essential in chronic human diseases and exhibit fewer side effects and more health benefits than chemically synthesized drugs [32]. SQS, a natural product, is a saponin. Notably, the therapeutic potential of saponins as anti-inflammatory agents has been reported in previously in vitro cell-based assays and in vivo mouse experiments. In LPS-induced RAW264.7 cells, saponins from Dioscorea nipponica Makino could substantially inhibit the production of NO, TNF-α, IL-1β, and IL-6. Moreover, saponins that were isolated from Madhuca longifolia seeds were found to exert marked anti-inflammatory activity in a mouse inflammatory model [33,34]. Additionally, we showed that SQS, a saponin from *C. oleifera*, could exert a significant inhibitory effect against inflammation by suppressing the activity of phosphorylase in NF-kB and MAPK signaling pathways, consequently inhibiting the production of pro-inflammatory factors and other effector molecules (IL-1β, IL-6, TNF-α, iNOS, and COX-2). Our findings, in conjunction with those reported previously regarding saponin-type natural compounds, highlight the potential of SQS as an anti-inflammatory agent (Figure 8).

## 4. Materials and Methods

### 4.1. Reagents

LPS was procured from Beijing Solaibao Technology Co., Ltd. (Beijing, China). The CCK-8 kit was purchased from Guangzhou Saiku Biological Technology Co., Ltd. (Guangzhou, China). The TNF-α, IL-6, and IL-1β ELISA kits were provided by Beijing 4A Biotech Co., Ltd. (Beijing, China). Primers for inducible COX-2, iNOS, TNF-α, IL-6, and IL-1β were obtained from QIAGEN Biotech Co., Ltd. (Beijing, China). Yishan Biotechnology Co., Ltd. (Shanghai, China) provided the RNA-Quick Purification kit. The SYBR Green Realtime PCR Master Mix was provided by TOYOBO Biotechnology Co., Ltd. (Shanghai, China). Antibodies against p-JNK (Thr183/Tyr185), p-pERK (Thr202/Tyr204), p-p38 (Thr180/Tyr182), p-IκBα (Ser32), and p-p65 (Ser468) were procured from Cell Signaling Technology (Danvers, MA, USA). Antibodies against c-Jun N-terminal kinase (JNK), extracellular signal-regulated kinases (ERK), p38, p65, IκBα, and β-actin were obtained from Proteintech (Wuhan, China).

### 4.2. Preparation of SQS

In brief, crushed camellia seeds were dried in an oven at 60 °C for 48 h, passed through a 60-mesh sieve, and extracted using a deep eutectic solvent comprising choline chloride and ethylene glycol (molar ratio of 1:3) with a water content of 70% (*v*/*v*) and a solid–liquid ratio of 1:35. Subsequently, ultrasonication was performed at 200 W for 20 min at room temperature. The supernatant was collected after centrifugation for further extraction. The supernatant was precipitated with 90% ethanol at 75 °C for 3 h thrice. The resulting supernatant was collected and concentrated in a rotary evaporator; D101 macroporous resin was used for adsorption, and elution was performed sequentially with deionized water, 60% ethanol, and 80% ethanol. The eluted solution was collected and concentrated using a rotary evaporator and lyophilized in a freeze-dryer. This process was repeated several times for dissolution, rotary evaporation, and lyophilization to obtain the SQS sample.

### 4.3. High-Performance Liquid Chromatography (HPLC) Analysis

The extracted SQS was dissolved in 70% methanol and filtered through a 0.45 μm nylon membrane filter before being dispensed in chromatographic vials for HPLC-DAD system (Agilent 1100, Agilent, Santa Clara, CA, USA) analysis. A Kromasil 100-5C18 UPLC column (250 × 4.6 mm, 1.8 µm) was used, and the column temperature was maintained at 30 °C. Mobile phase A was water, and mobile phase B was acetonitrile. The following linear gradients were used: 0–3.0 min (5% B), 3.0–8.0 min (5–20% B), and 8.0–20.0 min (20–5% B). The flow rate was 1 mL/min, the injector volume of the sample was 10 µL, and the detection wavelength was set at 215 nm.

### 4.4. Cell Culture

RAW-264.7 cells were purchased from the National Authentication Cell Bank (Shanghai, China). The RAW264.7 cells were maintained in DMEM supplemented with 10% fetal bovine serum and 1% penicillin-streptomycin and incubated at 37 °C in a 5% CO_2_ incubator. SQS was dissolved in DMEM with no more than 5% dimethyl sulfoxide (DMSO). The final concentration of SQS ranged from 0 to 30 μg/mL. Experiments were performed using RAW-264.7 cells within 10 passages.

### 4.5. Analysis of Cell Viability

Cell viability and cytotoxicity were measured using the CCK-8 kit. RAW-264.7 cells in the exponential growth phase were seeded at a density of 1 × 10^4^ cells/well in 96-well plates. After incubation at 37 °C for 6 h, different concentrations of SQS (10, 20, 30, 40, 50 μg/mL) were added and incubated for 24 h. Thereafter, 10 μL of CCK-8 solution was added to each well and incubated for 1 h. The absorbance was measured at 450 nm using a microplate reader (Synergy HTX, BIOTEX, Houston, TX, USA). Wells without cells were used as blanks and subtracted from each sample as background. The results are expressed as a percentage of the control cells.

### 4.6. Determination of Intracellular ROS Production

Intracellular ROS levels were quantified using an ROS-sensitive fluorescence indicator, DCFH-DA. Briefly, RAW 264.7 cells were seeded at a density of 1 × 10^5^ cells/mL in a 96-well plate, pre-treated with isolated SQS for 1 h, and stimulated with 1 µg/mL LPS for 3 h. Thereafter, the cells were washed thrice with 1 × washing buffer and loaded with 10 µM of the DCFH-DA detection reagent. After incubating in the dark for 30 min at 37 °C, cells were washed thrice with 1 × washing buffer. The DCF fluorescent were measured using a Fluorescence reader (Synergy HTX, BIOTEX, Houston, TX, USA) equipped with a 488/525 nm excitation/emission filter.

### 4.7. Reverse Transcription–Quantitative Polymerase Chain Reaction (RT-qPCR)

RAW-264.7 cells were seeded in 12-well plates at a density of 1 × 10^6^ cells/well, incubated for 12 h, and pre-treated with SQS or DMSO for 1 h before adding LPS (1 μg/mL) for cell stimulation. Total RNA from cells was extracted using an RNA-Quick Purification kit. According to the TOYOBO reverse transcription kit instructions, an appropriate amount of RNA was reverse-transcribed into cDNA. SYBR Green PCR Master Mix was used to detect mRNA levels, with GAPDH as the endogenous reference gene for IL-1β, TNF-α, IL-6, iNOS, and COX-2. RT-qPCR was performed using a real-time thermal cycler QuantStudio 6 Flex (QuantStudio 6 Flex, Thermo Fisher, Waltham, MA, USA) according to the manufacturer’s instructions. The relative expression levels were calculated using the 2^−ΔΔCt^ method. The oligonucleotide primers used for polymerase chain reaction amplification are shown in Table 1.

### 4.8. Enzyme-Linked Immunosorbent Assay (ELISA)

ELISA kits were used to detect the inhibitory effects of SQS on the production of pro-inflammatory cytokines. Briefly, RAW-264.7 cells were seeded at a density of 1 × 10^6^ cells/well in 12-well plates and incubated at 37 °C and 5% CO_2_ for 12 h. The complete medium was discarded, and cells were washed thrice with phosphate-buffered solution (PBS), followed by the addition of DMEM medium. Cells were then pre-treated with different concentrations of SQS for 1 h. After treatment with LPS (1 μg/mL) for different durations, the culture medium was centrifuged at 1500 rpm for 5 min. ELISA kits were used to assess the production of cytokines (IL-1β, IL-6, and TNF-α) in the collected supernatant. Optical density was then measured at 450 nm using a microplate reader (Synergy HTX, BIOTEX, Houston, TX, USA).

### 4.9. Western Blot Analysis

Briefly, RAW 264.7 cells (1 × 10^6^ cells/well) were incubated in 12-well plates for 12 h. Cells were then pre-treated with different concentrations of SQS for 1 h and stimulated with LPS (1 µg/mL) for 3 h. Cells were washed once with PBS and lysed with RIPA buffer. After lysis, the cell lysate was centrifuged at 12,000× *g* for 15 min at 4 °C. Protein quantification was performed using the BCA assay kit; proteins were mixed with 5X sample buffer, denatured by heating at 100 °C for 5 min, separated by performing 10% sodium dodecyl sulfate-polyacrylamide gel electrophoresis, and transferred onto a PVDF membrane (Immun-Blot PVDF, 0.2 µM). Subsequently, the membrane was blocked with protein-free rapid blocking reagent at room temperature for 10 min and incubated with primary antibodies against p65, p-p65, IκBα, p-IκBα, p-p38, p38, p-JNK, JNK, p-ERK 1/2, ERK 1/2, and β-actin (diluted 1:1000) overnight at 4 °C after washing once with 1X TBST. The membrane was then washed thrice with 1X TBST and incubated with HRP-conjugated secondary antibodies at room temperature for 2 h. Thereafter, the membrane was washed three more times with 1X TBST. Subsequently, the protein bands were visualized using an enhanced chemiluminescence reagent and chemiluminescence detection system (Thermo Scientific, Waltham, MA, USA). The protein bands were quantified using ImageJ (https://imagej.net/software/fiji/downloads, accessed on 4 October 2023) software (National Institutes of Health, Bethesda, Rockville, MD, USA).

### 4.10. Statistical Analysis

All measured data were obtained from at least three replicates and expressed as mean ± standard deviation (SD). The results of SQS time-dependent inhibition of transcription and expression of pro-inflammatory cytokines in LPS-stimulated RAW 264.7 cells were analyzed using two-way ANOVA, the other data analyses were performed using one-way analysis of variance (ANOVA), and the differences between groups were deemed statistically significant at *p* < 0.05. All statistical analyses were analyzed using GraphPad prismPrism 8.0.2 (GraphPad Software, Inc., SanDiego, CA, USA).

## 5. Conclusions

Herein, SQS that was extracted using deep eutectic solvents exhibited substantial anti-inflammatory effects. Following LPS-induced inflammation, treatment with SQS could markedly reduce ROS secretion in LPS-induced RAW264.7 macrophages and decrease the inflammatory response in a concentration-dependent manner, consequently reducing the production of COX-2, iNOS, IL-6, TNF-a, and IL-1β. The anti-inflammatory activity of SQS is potentially mediated via the suppression of ERK and JNK phosphorylation in the MAPK signal pathway and p65 and IκBα phosphorylation in the NF-κB signal pathway. Therefore, SQS can be developed as a potential therapeutic agent to treat inflammation-related diseases. Importantly, our study provides a theoretical basis for the potential application of SQS in medicine and biological products.

## Figures and Tables

**Table 1:** Oligonucleotide primer sequences used for reverse transcription–quantitative polymerase chain reaction.

Primer Name	Sequence (5′ to 3′)
GAPDH-F	GTGGCAAAGTGGAGATTGTTG
GAPDH-R	CTCCTGGAAGATGGTGATGG
IL-6-F	CTGCAAGAGACTTCCATCCAG
IL-6-R	AGTGGTATAGACAGGTCTGTTGG
IL-1β-F	GAAATGCCACCTTTTGACAGTG
IL-1β-R	TGGATGCTCTCATCAGGACAG
TNF-α-F	CTGAACTTCGGGGTGATCGG
TNF-α-R	GGCTTGTCACTCGAATTTTGAGA
iNOS-F	GGA GCG AGT TGT GGA TTG TC
iNOS-R	GTG AGG GCT TGG CTG AGT GAG
COX-2-F	GAA GTC TTT GGT CTG GTG CCT G
COX-2-R	GTC TGC TGG TTT GGA ATA GTT GC

## Data Availability

The data are available from the corresponding author upon reasonable request.

## Abbreviations

sasanquasaponin, SQS; lipopolysaccharide, LPS; nuclear factor-κB, NF-κB; reactive oxygen species, ROS; nitric oxide synthase, iNOS; cyclooxygenase-2, COX-2; tumor necrosis factor-α, TNF-α; interleukin-1β, IL-1β; interleukin-6, IL-6; prostaglandin E2, PGE_2_; c-Jun N-terminal kinase, JNK; extracellular signal-regulated kinases, ERK; high-performance liquid chromatography, HPLC; reverse transcription polymerase chain reaction, RT-PCR; enzyme-linked immunosorbent assay, ELISA; standard deviation, SD; analysis of variance, ANOVA; toll-like receptor 4, TLR-4; nitric oxide, NO; nuclear localization signal, NLS.
